# Supplementary material for: A Global Screen for Assembly State Changes of the Mitotic Proteome by SEC-SWATH-MS
Source: Cell Syst. 2020 Feb 26;10(2):133–155.e6. doi: 10.1016/j.cels.2020.01.001 (PMC7042714; doi:10.1016/j.cels.2020.01.001)

O60287 | NPA1P_HUMAN | URB1 C21orf108 KIAA0539 NOP254 NPA1
Monomer MW [kDa]: 254.389 Monomer expected elution fraction: 32

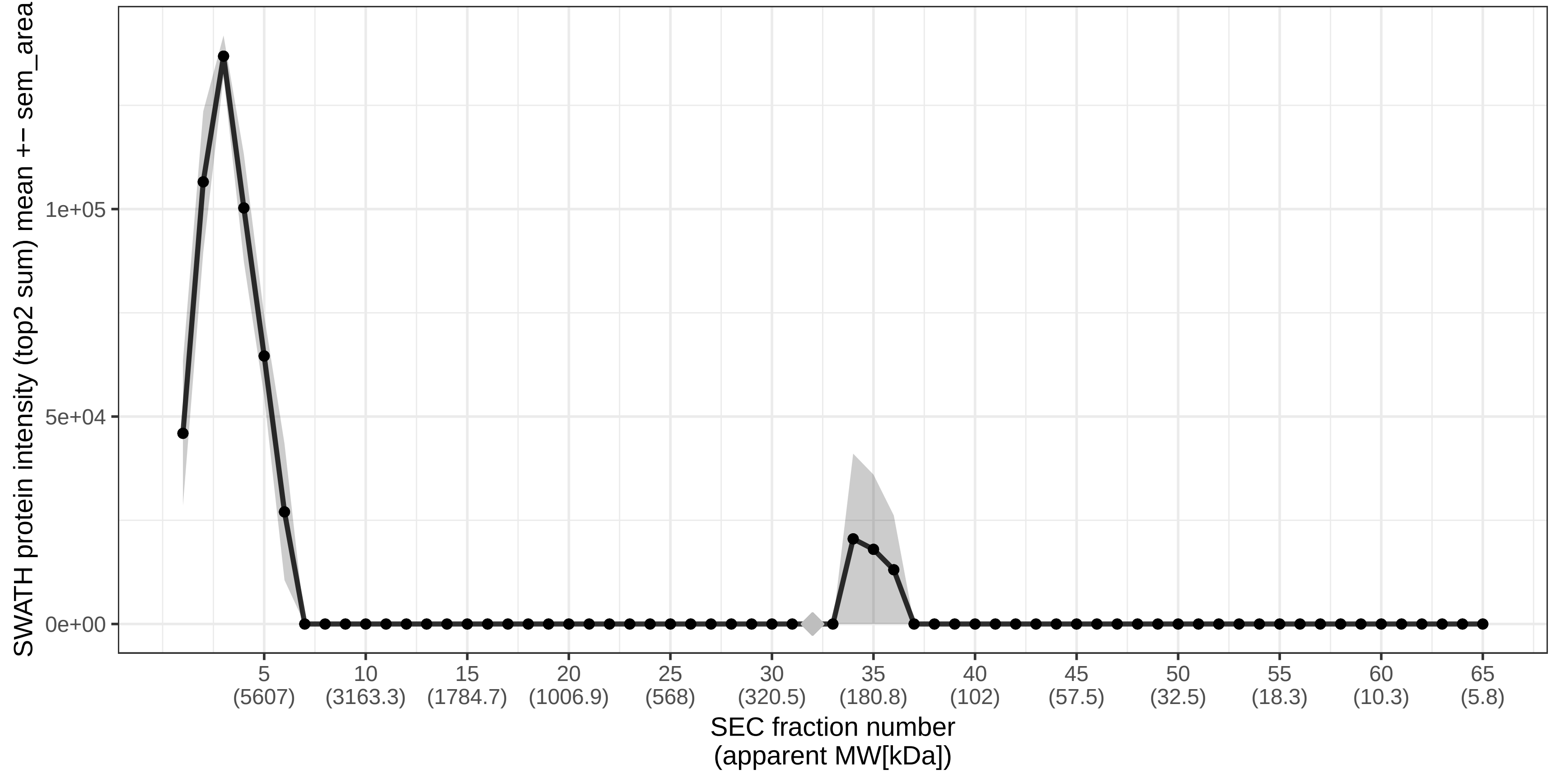

Supplement: Data S1. SEC-SWATH-MS Protein Chromatograms, Related to Figure 1 [file mmc6.zip › SECchrom_O60287_NPA1P_HUMAN_URB1_C21orf108_KIAA0539_NOP254_.pdf]
